# Activity of temocillin against ESBL-, AmpC-, and/or KPC-producing *Enterobacterales* isolated in Poland

**DOI:** 10.1007/s10096-020-03844-5

**Published:** 2020-02-24

**Authors:** Alicja Kuch, Bartłomiej Zieniuk, Dorota Żabicka, Sebastien Van de Velde, Elżbieta Literacka, Anna Skoczyńska, Waleria Hryniewicz

**Affiliations:** 1 Department of Epidemiology and Clinical Microbiology, National Medicines Institute, Chełmska 30/34, 00-725 Warsaw, Poland; 2 Eumedica S.A., Chemin de Nauwelette 1, 7170 Manage, Belgium

**Keywords:** Temocillin, *Enterobacterales*, ESBL/AmpC, KPC

## Abstract

We evaluated the in vitro effectiveness of temocillin and several commonly used antimicrobials against *Enterobacterales* bacteria in isolates from Polish patients. We tested 400 isolates: 260 extended-spectrum β-lactamase (ESBL)- and/or ampC β-lactamase (AmpC)-producing isolates; 40 *Klebsiella pneumoniae* carbapenemase (KPC)-producing isolates; and 100 ESBL-, AmpC-, and KPC-negative isolates. The minimal inhibitory concentrations (MICs) of temocillin and 16 other antimicrobials were determined by reference microdilution. We also determined the activities of fosfomycin and ceftazidime/avibactam in KPC-producing isolates. The antibiotic sensitivities were interpreted according to EUCAST, BSAC, and CLSI criteria. Overall, 91% of the isolates were susceptible to temocillin using the urinary tract infection breakpoint (≤ 32 mg/L), and 61.8% were susceptible using the systemic infection breakpoint (≤ 8 mg/L). Meropenem and imipenem were the most active drugs (MIC_50_ values of 0.06 and 0.5 mg/L, respectively). Colistin and ertapenem (both MIC_50_ = 0.12 mg/L) were less active than meropenem or imipenem, but some strains were 77% susceptible to each of them. Among the KPC-producing isolates, 42.5% had MIC values of ≤ 32 mg/L (urinary tract infection breakpoint), but 100% were resistant to temocillin (systemic infection breakpoint). Ceftazidime/avibactam was active against 100% of the KPC-producing isolates, and fosfomycin was active against 40%. The empirical susceptibility rate observed among the urinary isolates suggests that temocillin may be considered as an alternative to carbapenems in the absence of KPC-producing bacteria. With regard to isolates from other sources, temocillin might be useful as a documented therapy agent or an empirical treatment in hospitals with a low prevalence of ESBL/AmpC-producing strains.

## Introduction

Multidrug-resistant *Enterobacterales* bacteria have become a serious global concern, with limited therapeutic options for their control [1]. To meet the current challenges, there is an urgent need to discover new antimicrobials, or to re-examine known compounds such as fosfomycin, polymyxins, and temocillin [2].

Temocillin is a β-lactamase-resistant penicillin. It is the 6-α-methoxy derivative of ticarcillin, and is resilient to all classical and extended-spectrum TEM, SHV, and CTX-M enzymes and AmpC β-lactamases. Temocillin is used in few Western Europe countries [3], and only limited data are available on temocillin susceptibility in Eastern Europe, where microbial resistance to many antimicrobials is prevalent. According to the most recent report from the European Centre for Disease Prevention and Control in Poland over 65% of *Klebsiella pneumoniae* isolates reported to the European Antimicrobial Resistance Surveillance Network (EARS-Net) in 2018 were resistant to extended-spectrum cephalosporins; this represents an increase compared to previous years [4].

The aim of the present study was to evaluate the in vitro activity of temocillin and compare it to the activities of commonly used antimicrobials in respect of a large collection of *Enterobacterales* bacteria, especially ESBL- and/or AmpC-producing strains isolated from Polish patients with various infections.

## Materials and methods

The non-duplicate, well-characterized, clinical *Enterobacterales* isolates used in the present study were collected during laboratory surveillance conducted by the National Reference Centre for Susceptibility Testing between January 2000 and 2017 (Table 1). All the isolates were sub-cultured from storage (at -70 °C) and reidentified prior to testing. ESBL production and AmpC expression were verified using a double-disk synergy test, as previously described [5]. *Klebsiella pneumoniae* carbapenemase (KPC) production was verified using the disk test combined with phenylboronic acid [6] followed by polymerase chain reaction (PCR) [7]. β-lactamase production was controlled by PCR using specific primers for *bla*_CTX-M-1_-, *bla*_SHV_-, *bla*_TEM_-, and *bla*_KPC_-like genes [8]. The *bla*_KPC_ gene amplicons were all digested using a restriction enzyme (*Rsa*I; Thermo Scientific, Vilnius, Lithuania), which allowed *bla*_KPC-2_- and *bla*_KPC-3_-like variants to be distinguished [9].

**Table 1:** Characterisation of the *Enterobacterales* strains tested

Variable	*Klebsiella* spp.^1^ (*n* = 196)	*Escherichia coli* (*n* = 81)	*Proteus* spp.^2^ (*n* = 38)	CESP^3^ (*n* = 83)	Other species^4^ (*n* = 2)	Total (*n* = 400)
non-ESBL/AmpC/KPC	38	38	8	16	0	100
AmpC	1	1	18	19	0	39
ESBL	88	40	8	39	2	177
CTX-M	71	31	7	31	2	142
SHV	9	4	1	6	0	20
CTX-M + SHV	8	0	0	1	0	9
TEM	0	3	0	1	0	4
CTX-M + TEM	0	2	0	0	0	2
ESBL/AmpC	34	0	4	6	0	44
CTX-M	27	0	4	6	0	37
CTX-M + SHV	7	0	0	0	0	7
KPC	35	2	0	3	0	40
KPC-2-like	11	2	0	3	0	16
KPC-3-like	24	0	0	0	0	24
urine	72	30	16	21	1	140
blood	33	8	3	8	0	52
other^5^	91	43	19	54	1	208

^1^*Klebsiella pneumoniae* (186 isolates); *Klebsiella oxytoca* (10 isolates)

^2^*Proteus mirabilis* (36 isolates); *Proteus penneri* (1 isolate); *Proteus vulgaris* (1 isolate)

^3^CESP: *Citrobacter* spp. (*C. freundii* (18 isolates), *C. braakii* (2 isolates)); *Enterobacter* spp. (*E. cloacae* (34 isolates), *E. aerogenes* (2 isolates), *E. amnigenus* (1 isolate)); *Serratia* spp. (*S. marcescens* (18 isolates)); *Morganella* spp. (*M. morganii* (5 isolates)); and *Providencia* spp. (*P. rettgeri* (3 isolates))

^4^*Kluyvera intermedia* (1 isolate); *Aeromonas sobria* (1 isolate)

^5^bronchial secretions; cerebrospinal fluid; peritoneal fluid; pleural fluid; pus; skin lesions; sputum; wounds

The minimal inhibitory concentrations (MICs) of temocillin and 16 other antibiotics listed in Table 2 were evaluated using the microdilution method according to standard ISO 20776-1. The MIC of ceftazidime/avibactam was determined only for KPC-producing strains using a MIC Test Strip (Liofilchem®, Roseto degli Abruzzi, Italy). The quality control strains used in the study were: *Escherichia coli* ATCC 25922, *Pseudomonas aeruginosa* ATCC 27853, and *Escherichia coli* mcr-1-producing strain. The data were interpreted using EUCAST guidelines, except for temocillin and cefoxitin, where BSAC and CLSI breakpoints were used, respectively [10–12].

**Table 2:** Results of antibiotic susceptibility evaluation of 400 *Enterobacterales* strains

Antimicrobial	MIC (mg/L)			Interpretation			MIC breakpoints (mg/L)			% of resistant isolates among				
	MIC range	MIC_50_	MIC_90_	S number (%)	I number (%)	R number (%)	S	I	R	Non ESBL/AmpC/KPC (*n* = 100)	AmpC (*n* = 39)	ESBL (*n* = 177)	ESBL/AmpC (*n* = 44)	KPC (*n* = 40)
temocillin urinary breakpoint	1–256	8	32	364 (91.0)	-	36 (9.0)	≤32	-	>32^1^	0	2.6	5.7	4.6	57.5
temocillin systemic breakpoint	1–256	8	32	247 (61.8)	-	153 (38.2)	≤8	-	>8^1^	1.0	25.6	38.4	77.3	100
amoxicillin-clavulanic acid	0.5–256	32	128	103 (25.8)	-	297 (74.2)	≤8	-	>8^2^	20	100	87	100	100
piperacillin-tazobactam	0.25–256	32	256	114 (28.5)	38 (9.5)	248 (62.0)	≤8	16	>16^2^	2	92.3	70.6	97.7	100
ticarcillin-clavulanic acid	0.5–256	256	256	45 (11.3)	21 (5.3)	334 (83.4)	≤8	16	>16^2^	34	100	100	100	100
cefoxitin	1–256	32	256	147 (36.8)	36 (9)	217 (54.2)	≤8	16	≥32^3^	14	100	45.2	100	100
cefotaxime	0.03–256	32	32	94 (23.5)	2 (0.5)	304 (76.0)	≤1	2	>2^2^	6	100	98.9	100	100
ceftazidime	0.06–32	32	32	105 (26.3)	35 (8.8)	260 (64.9)	≤1	2–4	>4^2^	5	94.9	75.7	100	100
cefatzidime-avibactam (only KPC)	0.38–8	1.5	4	40 (100)	-	0 (0)	≤8	-	>8^2^	-	-	-	-	0
cefepime	0.015–16	16	16	120 (30.0)	22 (5.5)	258 (64.5)	≤1	2–4	>4^2^	0	41	89.3	100	100
ertapenem	0.0037–4	0.12	4	309 (77.0)	21 (5.3)	70 (17.7)	≤0.5	1	>1^2^	0	12.8	5.1	36.4	100
imipenem	0.06–32	0.5	8	326 (81.5)	38 (9.5)	36 (9.0)	≤2	4–8	>8^2^	0	0	0	0	90
meropenem	0.016–16	0.06	8	355 (88.8)	8 (1.9)	37 (9.3)	≤2	4–8	>8^2^	0	0	0	0	92.5
ciprofloxacin	0.0037–32	4	4	150 (37.5)	16 (4)	234 (58.5)	≤0.25	0.5	>0.5^2^	9	43.6	66.1	95.5	97.5
amikacin	0.5–32	8	32	229 (57.3)	48 (12)	123 (30.7)	≤8	16	>16^2^	1	41	35.6	40.9	64.1
gentamicin	0.25–256	32	256	172 (43.0)	5 (1.2)	223 (55.8)	≤2	4	>4^2^	1	71.8	79.1	90.9	35
tigecycline	0.12–16	2	8	162 (40.5)	106 (26.5)	132 (33.0)	≤1	2	>2^2^	16	74.4	32.8	38.6	25
colistin	0.03–16	0.12	16	308 (77.0)	-	92 (23.0)	≤2	-	>2^2^	13	58.9	19.8	43.2	5
fosfomycin (only KPC)	1 - >256	64	256	16 (40.0)	–-	24 (60.0)	≤32	-	>32^2^	-	-	-	-	60
trimethoprim/ sulfamethoxazole	0.06–32	32	32	130 (32.5)	5 (1.3)	265 (66.2)	≤2	4	>4^2^	18	66.7	84.8	86.4	82.5

MIC, minimum inhibitory concentration; MIC_50_ and MIC_90_, MIC for 50% and 90% of the isolates, respectively

^1^British Society for Antimicrobial Chemotherapy (BSAC)

^2^European Committee on Antimicrobial Susceptibility Testing (EUCAST), Clinical breakpoints for bacteria, Version 9.0; 2019-01-01

^3^Clinical and Laboratory Standards Institute (CLSI). Performance standards for antimicrobial susceptibility testing, Document M100-29 (29th edition)

## Results

The 400 isolates tested were recovered from urine (35%), blood (13%), and other clinical specimens (52%; from bronchial secretions, cerebrospinal fluid, peritoneal fluid, pleural fluid, pus, skin lesions, sputum, and wounds) (Table 1). With regard to the resistant phenotypes, isolates producing ESBLs (CTX-M, SHV, or TEM) and/or isolates with acquired or overexpressed AmpC-type β-lactamase were the most prevalent (*n* = 260, 65%), followed by KPC-producing isolates (*n* = 40, 10%), mostly represented by KPC-like-3-positive bacteria (*n* = 24, 60%) (Table 1).

The results of a detailed analysis of the susceptibility data are shown in Table 2. The MICs of temocillin ranged from 1 to 256 mg/L. Most of the isolates (*n* = 364, 91%) had an MIC value between 4 and 32 mg/L (MIC_50_ and MIC_90_ values of 8 and 32 mg/L, respectively) (Table 2). Overall, 91% of the isolates were susceptible to temocillin according to the BSAC urinary breakpoint (≤ 32 mg/L), and 61.7% were susceptible according to the systemic breakpoint. Temocillin was very effective against all the species tested when the urinary breakpoint was used (Table 3). According to the systemic breakpoint, temocillin was less effective against *Klebsiella* and CESP spp. (*Citrobacter*, *Enterobacter*, *Serratia*, *Providencia*, *Morganella*, and *Hafnia* that produced inducible chromosomally encoded AmpC-type) with resistance values of 53.1% and 44.6%, respectively. Among the CESP members, the most resistant species were *Serratia marcescens* (88.9%) and *Enterobacter* spp. (35.1%) (data not shown).

**Table 3:** Results of temocillin susceptibility evaluation of 400 *Enterobacterales* strains

	MICs for TEM			Resistance rate to temocillin (% of TEMu / % of TEMs) among:											
	MIC range	MIC_50_	MIC_90_	All isolates	Non-ESBL/AmpC/ KPC (*n* = 100)	AmpC (*n* = 39)	ESBL (CTX-M) (*n* = 142)	ESBL (SHV) (*n* = 20)	ESBL (CTX-M/ SHV) (*n* = 9)	ESBL (TEM) (*n* = 4)	ESBL (CTX-M/ TEM) (*n* = 2)	ESBL (CTX-M)/AmpC (*n* = 37)	ESBL (CTX-M/SHV)/AmpC (*n* = 7)	KPC-2 (*n* = 16)	KPC-3 (*n* = 24)
*Klebsiella* spp.^1^ (*n* = 196)	1–256	16	64	12.7/53.1	0/0	0/2.5	2.1/**23.9**	0/**10**	0/11.1	0/0	0/0	**2.7/62.2**	**14.3/14.3**	**25/68.8**	**66.7/100**
*Escherichia coli* (*n* = 81*)*	2–128	8	16	3.6/13.6	0/1	0/0	0.7/3.5	0/0	0/0	**25/50**	0/**50**	0/0	0/0	6.3/12.5	0/0
*Proteus* spp.^2^ (*n* = 28)	1–16	2	4	0/2.6	0/0	0/0	0/0	0/5	0/0	0/0	0/0	0/0	0/0	0/0	0/0
CESP^3^ (*n* = 83)	2–256	8	32	9.6/44.6	0/0	**2.6/23.1**	**2.8/**10.6	0/**15**	**11.2/33.3**	0/25	0/0	0/13.5	0/0	12.5/18.8	0/0

MIC, minimum inhibitory concentration; MIC_50_ and MIC_90_, MIC for 50% and 90% of the isolates respectively

TEMu – resistant rate to temocillin at urinary breakpoint; TEMs – resistant rate to temocillin at systemic breakpoint

^1^*Klebsiella pneumoniae* (186 isolates), *Klebsiella oxytoca* (10 isolates)

^2^*Proteus mirabilis* (36 isolates), *Proteus penneri* (1 isolate), *Proteus vulgaris* (1 isolate)

^3^CESP: *Citrobacter* spp. (*C. freundii* (18 isolates), *C. braakii* (2 isolates)); *Enterobacter* spp. (*E. cloacae* (34 isolates), *E. aerogenes* (2 isolates), *E. amnigenus* (1 isolate)); *Serratia* spp. (*S. marcescens* (18 isolates)); *Morganella* spp. (*M. morganii* (5 isolates)]; and *Providencia* spp. (*P. rettgeri* (3 isolates))

Boldface indicates the highest level of resistance among the groups of isolates

According to the urinary breakpoint, among the ESBL- and/or AmpC-producing isolates, the temocillin susceptibility rates were between 94% and 97%. According to the systemic breakpoint, the susceptibility rates were 22.7% for ESBL- and AmpC-producers, 61.6% for ESBL-producers, and 74.4% for AmpC-producers (Table 2).

The KPC producers were resistant to several antimicrobials. These isolates were all resistant to temocillin according to the systemic infection breakpoint, but only 42.5% were susceptible according to the urinary tract infection breakpoint. Ceftazidime/avibactam (100% susceptibility) and colistin (95% susceptibility) were the most effective against KPC-producing isolates.

## Discussion

Temocillin has been used for several years in some European countries, and is approved for the treatment of septicaemia, and urinary tract and lower respiratory tract infections [13–16]. However, it is still not available in Poland. Therefore, in the present analysis we assessed the prevalence of susceptibility to temocillin in *Enterobacterales* bacteria before it becomes available in our country.

There are currently no EUCAST breakpoints for temocillin [10]. Therefore, *Enterobacterales* bacteria are categorized as susceptible at MIC values of 8, 16, or 32 mg/L, depending on the country [14]. In the present study, we used BSAC [11] clinical breakpoints with criteria established separately for systemic (8 mg/L) and urinary tract (32 mg/L) infections. The temocillin MIC_50_ and MIC_90_ values for the total set of isolates calculated according to the urinary breakpoint were higher than those reported by Alexandre et al. [14] for urinary tract infection (UTI) isolates (8 and 32 mg/L versus 3 and 6 mg/L, respectively). However, our set of isolates comprised both UTI cases and isolates from other sites of infection. When MICs were calculated only for isolates from urine, the MIC_50_ and MIC_90_ values were similar (data not shown), suggesting a possible role for temocillin in the treatment of urinary tract infections in Poland, regardless of the clinical breakpoint used. As expected, based on the literature non-ESBL/AmpC/KPC-producing isolates were very susceptible (100% and 99%), regardless of the clinical breakpoint used [15–18].

With regard to bacterial species tested and according to the urinary breakpoint, temocillin retained a high level of activity against all bacterial species producing ESBL and/or AmpC enzymes. However, when the systemic infection breakpoint was applied, only *E. coli* and *Proteus* spp. species remained susceptible, suggesting a possible role for temocillin in the treatment of urinary tract infections due to those two species [19]. More generally, temocillin was highly effective against most *Enterobacterales* bacteria, especially when the urinary tract infection breakpoint was used, and was least active against *K. pneumoniae*. The second most temocillin-resistant species was *S. marcescens*, with 77.8% of resistant isolates harbouring CTX-M enzymes (data not shown).

The majority of ESBL- or ESBL/AmpC-producers were susceptible using the urinary breakpoint but the resistance rate increased significantly using the systemic breakpoint. Previously, Rodriguez-Villalobos et al. [18] and Kresken et al. [20] pointed out that CTX-M-15 producers were less frequently susceptible to temocillin than other CTX-M-type-producing isolates. In Poland, as in other European countries, the population of *Enterobacterales* bacteria has been dominated by CTX-M-15 producers [21].

With regard to *K. pneumoniae*, the temocillin-resistant strains were dominated by CTX-M- and KPC-producing isolates, regardless of the sample origin (data not shown). Contrary to the previous reports by Adams-Haduch et al. [22] and Woodford et al. [23], we were unable to confirm the susceptibility to temocillin among KPC-producing isolates. In the present study, 100% and over 50% of the KPC-producers were resistant to temocillin according to the systemic and urinary breakpoints, respectively. Our data are however in line with a report from Greece describing low temocillin activity against KPC-producers: 97.3% and 42% were resistant according to systemic and urinary breakpoints, respectively [24]. The low number of KPC-producing isolates is a limitation of the study, but the set of clinical isolates tested was representative of the population of KPC-producing isolates cultured from clinical specimens between 2010 and 2017 in Poland. However, among our KPC-producers, 20% had CTX-M enzymes and this could already be a reason why they are more resistant to temocillin. It would be of interest to further investigate the presence of other resistance mechanisms such as upregulated efflux or permeability for instance.

The present study is the first Polish evaluation of the in vitro susceptibility of *Enterobacterales* isolates to temocillin. It demonstrated the effectiveness of the antibiotic against a collection of tested microbes, especially when the urinary breakpoint was used. In contrast with previously published data, our study did not confirm the susceptibility of KPC-producing isolates to temocillin.
